# Li_2_^100depl^MoO_4_ Scintillating Bolometers for Rare-Event Search Experiments

**DOI:** 10.3390/s23125465

**Published:** 2023-06-09

**Authors:** Iulian C. Bandac, Alexander S. Barabash, Laurent Bergé, Yury A. Borovlev, José Maria Calvo-Mozota, Paolo Carniti, Maurice Chapellier, Ioan Dafinei, Fedor A. Danevich, Louis Dumoulin, Federico Ferri, Andrea Giuliani, Claudio Gotti, Philippe Gras, Veronika D. Grigorieva, Aldo Ianni, Hawraa Khalife, Vladislav V. Kobychev, Sergey I. Konovalov, Pia Loaiza, Madhujith Madhukuttan, Evgeny P. Makarov, Pierre de Marcillac, Stefanos Marnieros, Claire A. Marrache-Kikuchi, Maria Martinez, Claudia Nones, Emiliano Olivieri, Alfonso Ortiz de Solórzano, Gianluigi Pessina, Denys V. Poda, Thierry Redon, Jean-Antoine Scarpaci, Vladimir N. Shlegel, Volodymyr I. Tretyak, Vladimir I. Umatov, Mykola M. Zarytskyy, Anastasiia Zolotarova

**Affiliations:** 1 Laboratorio Subterráneo de Canfranc, 22880 Canfranc-Estación, Spain; ibandac@lsc-canfranc.es (I.C.B.); jmcalvo@lsc-canfranc.es (J.M.C.-M.); 2 National Research Centre Kurchatov Institute, Kurchatov Complex of Theoretical and Experimental Physics, 117218 Moscow, Russia; barabash@itep.ru (A.S.B.); konovalov@itep.ru (S.I.K.); yumatov@itep.ru (V.I.U.); 3 Université Paris-Saclay, CNRS/IN2P3, IJCLab, F-91405 Orsay, France; laurent.berge@ijclab.in2p3.fr (L.B.); maurice.chapellier@ijclab.in2p3.fr (M.C.); louis.dumoulin@ijclab.in2p3.fr (L.D.); andrea.giuliani@ijclab.in2p3.fr (A.G.); pia.loaiza@ijclab.in2p3.fr (P.L.); madhukuttan@ijclab.in2p3.fr (M.M.); pierre.de-marcillac@ijclab.in2p3.fr (P.d.M.); stefanos.marnieros@ijclab.in2p3.fr (S.M.); claire.marrache@ijclab.in2p3.fr (C.A.M.-K.); emiliano.olivieri@ijclab.in2p3.fr (E.O.); thierry.redon@ijclab.in2p3.fr (T.R.); jean-antoine.scarpaci@ijclab.in2p3.fr (J.-A.S.); 4 Nikolaev Institute of Inorganic Chemistry, 630090 Novosibirsk, Russia; yubor@ngs.ru (Y.A.B.); grigoryeva@niic.nsc.ru (V.D.G.); maccarov@niic.nsc.ru (E.P.M.); shlegel@niic.nsc.ru (V.N.S.); 5 Escuela Superior de Ingeniería y Tecnología, Universidad Internacional de La Rioja, 26006 Logroño, Spain; 6 INFN, Sezione di Milano Bicocca, I-20126 Milano, Italy; paolo.carniti@mib.infn.it (P.C.); claudio.gotti@mib.infn.it (C.G.); pessina@mib.infn.it (G.P.); 7 INFN, Sezione di Roma, I-00185 Rome, Italy; ioan.dafinei@roma1.infn.it; 8 Institute for Nuclear Research of NASU, 03028 Kyiv, Ukraine; danevich@kinr.kiev.ua (F.A.D.); kobychev@kinr.kiev.ua (V.V.K.); tretyak@kinr.kiev.ua (V.I.T.); m.zarytskyy@kinr.kiev.ua (M.M.Z.); 9 INFN Sezione di Roma Tor Vergata, I-00133 Rome, Italy; 10 IRFU, CEA, Université Paris-Saclay, F-91191 Gif-sur-Yvette, France; federico.ferri@cern.ch (F.F.); philippe.gras@cern.ch (P.G.); hawraa.khalife@cea.fr (H.K.); claudia.nones@cea.fr (C.N.); anastasiia.zolotarova@cea.fr (A.Z.); 11 INFN, Laboratori Nazionali del Gran Sasso, I-67100 Assergi, Italy; aldo.ianni@lngs.infn.it; 12 Centro de Astropartículas y Física de Altas Energías, Universidad de Zaragoza, 50009 Zaragoza, Spain; mariam@unizar.es (M.M.); alfortiz@unizar.es (A.O.d.S.); 13 ARAID Fundación Agencia Aragonesa para la Investigación y el Desarrollo, 50018 Zaragoza, Spain

**Keywords:** cryogenic detector, bolometer, crystal scintillator, lithium molybdate, molybdenum depleted in ^100^Mo, rare events

## Abstract

We report on the development of scintillating bolometers based on lithium molybdate crystals that contain molybdenum that has depleted into the double-β active isotope ${}^{100}$Mo (Li${}_{2}$${}^{100\mathrm{depl}}$MoO${}_{4}$). We used two Li${}_{2}$${}^{100\mathrm{depl}}$MoO${}_{4}$ cubic samples, each of which consisted of 45-millimeter sides and had a mass of 0.28 kg; these samples were produced following the purification and crystallization protocols developed for double-β search experiments with ${}^{100}$Mo-enriched Li${}_{2}$MoO${}_{4}$ crystals. Bolometric Ge detectors were utilized to register the scintillation photons that were emitted by the Li${}_{2}$${}^{100\mathrm{depl}}$MoO${}_{4}$ crystal scintillators. The measurements were performed in the CROSS cryogenic set-up at the Canfranc Underground Laboratory (Spain). We observed that the Li${}_{2}$${}^{100\mathrm{depl}}$MoO${}_{4}$ scintillating bolometers were characterized by an excellent spectrometric performance (∼3–6 keV of FWHM at 0.24–2.6 MeV γs), moderate scintillation signal (∼0.3–0.6 keV/MeV scintillation-to-heat energy ratio, depending on the light collection conditions), and high radiopurity (${}^{228}$Th and ${}^{226}$Ra activities are below a few µBq/kg), which is comparable with the best reported results of low-temperature detectors that are based on Li${}_{2}$MoO${}_{4}$ using natural or ${}^{100}$Mo-enriched molybdenum content. The prospects of Li${}_{2}$${}^{100\mathrm{depl}}$MoO${}_{4}$ bolometers for use in rare-event search experiments are briefly discussed.

## 1. Introduction

Crystal scintillators are widely used in searches for rare-event processes (such as two-ν and ν-less double-β decays, rare α and β decays, and dark matter particles), particularly in the technologies of low-temperature detectors [1,2,3,4,5]. Among them, molybdenum that contains crystals represents a long-standing interest for double-β decay searches [1,2,3,4,5], mainly in the isotope form of ${}^{100}$Mo (possible also for ${}^{92}$Mo and ${}^{98}$Mo [6]); this isotope is also promising for solar and supernova neutrino detection [7,8,9,10]. Different compounds with natural molybdenum content, as well as a few having ${}^{100}$Mo-enriched molybdenum content, have been developed and investigated as low-temperature detectors with the property of simultaneous heat and scintillation detection, i.e., scintillating bolometers [1,2,3,4,5]. Lithium molybdate (Li${}_{2}$MoO${}_{4}$) has been found to be one of the most promising Mo-containing scintillators for such applications [11,12,13]. Natural and ${}^{100}$Mo-enriched Li${}_{2}$MoO${}_{4}$ (Li${}_{2}$${}^{100}$MoO${}_{4}$; enrichment is ∼97%) have been developed within the LUMINEU project and have been used in searches for ${}^{100}$Mo double-β decay [13,14,15,16]. The LUMINEU technology of scintillating bolometers has been adopted for the CUPID-Mo double-β experiments [17,18,19,20]. Such a detector material has also been selected for the CUPID [21,22,23,24,25] and CROSS [23,25,26,27] projects, and it garners great interest for use in the AMoRE experiment [28,29,30,31,32].

Lithium is also an element that has had a long-standing interest for use in rare-event search experiments. The dominant isotope, i.e., ${}^{7}$Li (92% of natural lithium), is a light nucleus with a non-zero spin, thus making it a viable candidate for probing spin-dependent dark matter interactions [33,34]. Moreover, ${}^{7}$Li is a good target for searching for solar axions via a resonant absorption of an axion by ${}^{7}$Li and its subsequent γ de-excitation [11,35,36,37,38]. Last but not least, presence of ${}^{6}$Li (8% of natural lithium) allows neutron detection via a ${}^{6}$Li(n, t)α reaction, which is characterized by a high cross-section to thermal neutrons; furthermore, enrichment in ${}^{6}$Li (of up to 95%) is feasible and can enhance the detection efficiency. Thus, Li-containing detectors are of special interest for neutron flux monitoring in rare-event search experiments [39,40].

In this work, we present the development and investigation of scintillating bolometers based on a new type of the Li${}_{2}$MoO${}_{4}$ compound, which is produced from molybdenum that is depleted in ${}^{100}$Mo (Li${}_{2}$${}^{100\mathrm{depl}}$MoO${}_{4}$). The low concentration of the double-β active isotope ${}^{100}$Mo (the transition energy is $Q_{\beta \beta }$ = 3084 keV; the half-life is ∼7 × 10${}^{18}$ yr [16,41]) significantly reduces the related background counting rate, which is on the level of 10 mBq/kg in a Li${}_{2}$${}^{100}$MoO${}_{4}$ crystal (an order of magnitude lower for the natural one), and it can be a dominant internal background of this material. Therefore, Li${}_{2}$${}^{100\mathrm{depl}}$MoO${}_{4}$ appears to be more suitable than natural or ${}^{100}$Mo-enriched Li${}_{2}$MoO${}_{4}$ crystals for dark matter and axion search experiments using ${}^{7}$Li, for double-β decay searches in ${}^{92}$Mo and ${}^{98}$Mo, and for ${}^{6}$Li-based neutron detection. Moreover, Li${}_{2}$${}^{100\mathrm{depl}}$MoO${}_{4}$ can be used in bolometric experiments to search for double-β decay in ${}^{100}$Mo as a complementary detector for better understanding the background model. Therefore, a goal of this work is to study the prospects of Li${}_{2}$${}^{100\mathrm{depl}}$MoO${}_{4}$ low-temperature detectors for rare-event search applications.

## 2. Development and Test of Li${}_{2}$${}^{100\mathrm{depl}}$MoO${}_{4}$ Scintillating Bolometers

### 2.1. Crystals Production and Construction of Detectors

In this study, we used two Li${}_{2}$${}^{100\mathrm{depl}}$MoO${}_{4}$ scintillation crystals with a size of 45 × 45 × 45 mm and a mass of around 0.28 kg each, which are identical to the Li${}_{2}$${}^{100}$MoO${}_{4}$ crystals that were produced for the CROSS experiment [26]. The samples were cut from the same crystal boule, which was grown using the low-thermal-gradient Czochralski technique as detailed in [42]. A 4N purity lithium carbonate, which was selected for the LUMINEU and CUPID-Mo crystals production [13,14,17], and molybdenum oxide that was depleted in ${}^{100}$Mo (∼0.01% of ${}^{100}$Mo; i.e., 1000 times lower than in natural Mo) have been used as the starting materials. The ${}^{100\mathrm{depl}}$MoO${}_{3}$ material purification, solid-phase synthesis of the Li${}_{2}$${}^{100\mathrm{depl}}$MoO${}_{4}$ compound, and crystal growth in a Pt crucible using the low-thermal-gradient Czochralski method (double crystallization approach) were realized while following the protocols of Mo-containing crystals production that was developed by LUMINEU [13,14,43] and adopted by CUPID-Mo [17]. A large crystal boule (the cylindrical part is around ⊘60 × 100 mm) has been grown to have a crystal yield of about 80% from the initial charge [42] and two twin cubic samples (with a few millimeter chamfers) were produced.

**Figure 1:**
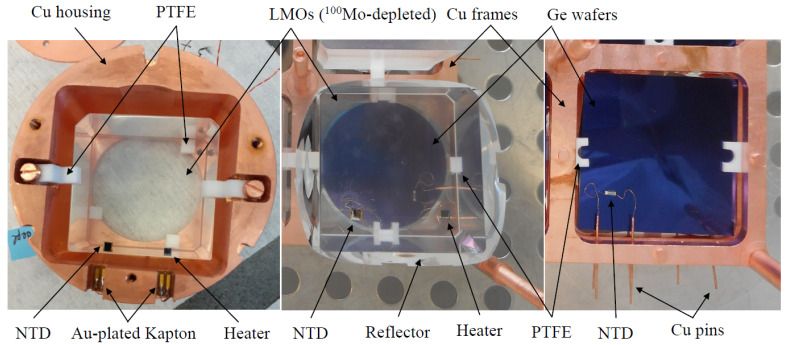
Photographs of Li${}_{2}$${}^{100\mathrm{depl}}$MoO${}_{4}$ low-temperature detectors LMO-depl-1 (**left**) and LMO-depl-2 (**middle**). Both crystals have two epoxy-glued sensors; the left one is an NTD Ge thermistor, while the right one is a P-doped Si heater. Each scintillator was accompanied by a circular bolometric Ge light detector, as can be seen in the transparent area of the crystal in the middle panel. An additional square-shaped Ge light detector (**right**) was used for the LMO-depl-2 sample. All light detectors were instrumented with an NTD Ge sensor.

The assembly of detectors have been carried out in an ISO class 4 clean room at IJCLab (Orsay, France). The first Li${}_{2}$${}^{100\mathrm{depl}}$MoO${}_{4}$ sample (LMO-depl-1), corresponding to the upper part of the boule, was mounted inside a Cu housing using polytetrafluoroethylene (PTFE) supporting elements and Cu screws, as shown in Figure 1 (left). The holder design is rather similar to one used in bolometric measurements, using the same size of Li${}_{2}$${}^{100}$MoO${}_{4}$ crystal [23] and slightly larger TeO${}_{2}$ sample [44]. The second Li${}_{2}$${}^{100\mathrm{depl}}$MoO${}_{4}$ sample (LMO-depl-2), corresponding to the bottom part of the boule, was assembled using Cu frames and columns and PTFE pieces, as seen in Figure 1 (middle). This sample was a part of a twelve-crystal array, in which all of the other crystals were Li${}_{2}$${}^{100}$MoO${}_{4}$ [25]. The lateral side of the LMO-depl-2 crystal was surrounded by a reflective film (Vikuiti™) to improve the light collection, while the Cu housing of the LMO-depl-1 detector served as a reflective cavity; however the aperture reduces the direct sight of the light detector. Thus, the light collection is sub-optimal both due to the poor reflectivity of copper and low scintillation photons collection efficiency of the light detector(s).

To detect particle interactions, each Li${}_{2}$${}^{100\mathrm{depl}}$MoO${}_{4}$ crystal was instrumented with a neutron transmutation-doped Ge [45] thermistor (NTD). A sensor with a size of 3 × 3 × 1 mm was epoxy-glued onto the crystal’s surface using six spots of a bi-component glue (Araldite^®^ Rapid). The temperature-dependent resistance of the NTDs can be approximated as $R\left(T\right)=R_{0}\cdot e^{{\left(T_{0}/T\right)}^{0.5}}$ using the parameters $R_{0}$∼ 1 Ω and $T_{0}$∼ 3.7 K. NTD sensors with similar irradiation parameters have been used in the CUPID-Mo experiment [17] and in CUPID-related R&D tests [22,23,24,25]. Moreover, a P-doped Si heater [46] was glued on each crystal using a veil of the epoxy glue. This heating element was exploited to be injected using the Jules effect power, which can be used for, e.g., the stabilization of the thermal gain [47], optimization of the detector working point, heating of a device if necessary (as in CUPID-0 [48]), or pile-up simulations [49]. To provide electrical contacts, the NTDs and heaters were wire-bonded using ⊘25 µm Au wires.

To allow for the detection of Li${}_{2}$${}^{100\mathrm{depl}}$MoO${}_{4}$ scintillation (with the emission maximum at ∼590 nm at low temperatures [12]), we accompanied crystals with bolometric detectors that were based on electronic-grade purity Ge wafers, which were supplied by Umicore (Belgium) [50]. Two of them had a circular shape (with a size of ⊘45 × 0.18 mm each), while the third device was square-shaped (45 × 45 × 0.30 mm). All of the Ge disks were coated on both sides with a 70 nm SiO layer, which was aimed at reducing the light reflection [17,48,51]. Smaller NTD sensors (3 × 1 × 1 mm or 3 × 0.7 × 1 mm) were attached to the Ge wafers using a veil of epoxy glue. The Ge disks were PTFE-clamped in the Cu structure. A single circular light detector (LD-1-c) was coupled to the LMO-depl-1 crystal, while both the circular (LD-2-c) and square-shaped (LD-2-s) bolometric photodetectors viewed the LMO-depl-2 crystal. The mounted Ge light detectors are shown in Figure 1 (middle and right).

### 2.2. Operation at Canfranc Underground Laboratory

**Figure 2:**
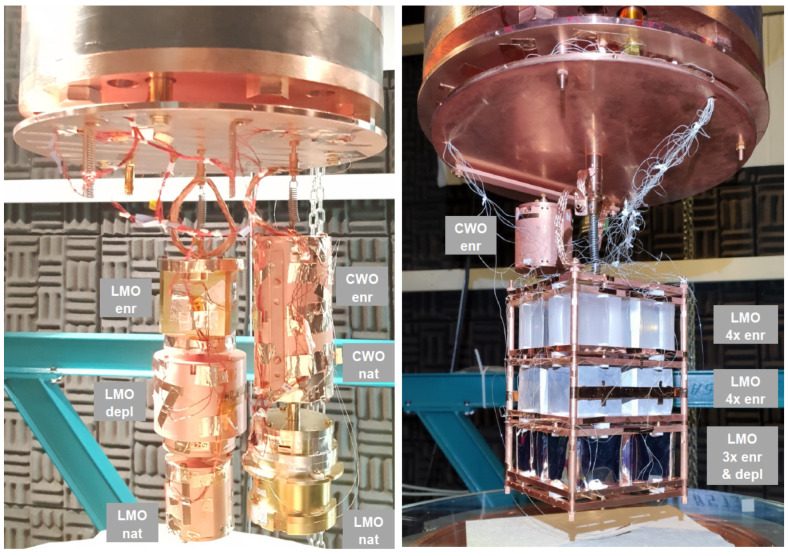
Detector configurations in the C2U cryogenic runs at the LSC, where the Li${}_{2}$${}^{100\mathrm{depl}}$MoO${}_{4}$ scintillating bolometers LMO-depl-1 (**left**) and LMO-depl-2 (**right**) were operated. Other scintillating bolometers are based on Li${}_{2}$MoO${}_{4}$ crystals with natural (CROSS [57] and CLYMENE [58,59] R&D) and ${}^{100}$Mo-enriched (joint CROSS and CUPID R&D [23,25]) molybdenum content, as well as natural and ${}^{116}$Cd-enriched CdWO${}_{4}$ crystals [52,60,61,62].

The Li${}_{2}$${}^{100\mathrm{depl}}$MoO${}_{4}$ scintillating bolometers were operated in the CROSS cryogenic set-up (C2U) [23,52] at the Canfranc Underground Laboratory (LSC, Spain), which provided a substantial reduction in the cosmic muon flux thanks to the rock overburden [53]. The detectors were assembled as parts of scintillating bolometer arrays and were installed inside the cryostat, as illustrated in Figure 2. The facility exploits the HEXA-DRY dilution fridge by CryoConcept (France), which is equipped with the Ultra-Quiet Technology™ [54] to decouple a pulse tube (Cryomech PT415) from the dilution unit, thus reducing vibrations [55]. To further improve the noise conditions, the detector arrays were spring-suspended from the cold plate of the cryostat. The detector volume inside the cryostat is shielded on top by a 13 cm thick disk made of interleaved lead and copper (partially seen in Figure 2), while the outer vacuum chamber is surrounded by a 25 cm thick layer of low-radioactivity lead. In addition, a deradonized air (∼1 mBq/m${}^{3}$ of Rn [56]) flow around the cryostat was supplied the whole time during the experiment with the LMO-depl-2 detector.

After reaching the base temperature of the cryostat (∼10 mK), we regulated the detector plate temperature at 18 mK and then 12 mK for measurements using the LMO-depl-1 bolometer; the plate temperature was regulated at 14 mK for the LMO-depl-2 bolometer operation. The control and readout of the bolometers was performed with the help of low-noise, room temperature, DC front-end electronics, which were restyled from the Cuoricino experiment [63]. The data acquisition (DAQ) system was composed of two 12-channel boards with an integrated 24-bit ADC and a programmable 6-pole Bessel–Thomson anti-aliasing filter (the cut-off frequency of the low-pass filter was set at 300 Hz) [64,65].

In order to find an optimal working point of the detectors that represents the best signal-to-noise ratio, we spanned the bolometric response with respect to the current across an NTD [66]. We used heaters to inject thermal pulses to both Li${}_{2}$${}^{100\mathrm{depl}}$MoO${}_{4}$ bolometers, while LED generated photons, which were transmitted from a room temperature LED (the emission maximum was at ∼880 nm) through an optic fiber, were exploited for the light detectors. The heater/LED signal injection was performed with the help of a wave function generator (Keysight 33500B). As a result of the optimization, we polarized the NTDs of the detectors under a current strength of a few nA, which reduced the NTD resistances from hundreds of MΩ (at low power) to a few MΩ (at the working point).

For each operational temperature, we performed measurements using a removable ${}^{232}$Th γ source, which was made of a thoriated tungsten wire; we also performed measurements without the source (data are referred to as the calibration and background, respectively). Even if the γ source was primarily conceived for the calibration of the Li${}_{2}$${}^{100\mathrm{depl}}$MoO${}_{4}$ bolometers, we also used it to evaluate the energy scale of the light detectors, similar to [17,44].

The continuous data of each channel were acquired at a sampling rate of 2 kS/s and stored on a disk for the offline analysis. We processed the data with the help of a MATLAB-based analysis tool [67], which implements the signal processing using the Gatti–Manfredi optimum filter [68] to maximize the signal-to-noise ratio. In order to apply the filter, we used data-based information about the signal shape (represented by an average signal of high-energy events in the order of tens) and measured noise (represented by 10,000 waveforms with no signal). The data were triggered using a threshold corresponding to 5σ of the filtered noise. For each triggered signal, we collected information about its amplitudes (i.e., energy) and about several pulse-shape parameters. The results of the detectors characterization are presented in the next section.

## 3. Characterization of Li${}_{2}$${}^{100\mathrm{depl}}$MoO${}_{4}$ Scintillating Bolometers

### 3.1. Performance of Detectors

At first, we investigated the recorded bolometric signals in terms of the time constants of the signal shape. The rising part of a signal is commonly characterized by the rise time parameter, which is computed as the time required by the signal to increase from 10% to 90% of its amplitude. The descending part is described by the decay time, which is defined here as the time required to drop from 90% to 30% of signal amplitude. The rise and decay time parameters of the operated low-temperature detectors are summarized in Table 1. We found that the Li${}_{2}$${}^{100\mathrm{depl}}$MoO${}_{4}$ bolometers have signals with a rise time of ∼20 ms and decay time of ∼100 ms. These time constants are similar to the values reported for NTD-instrumented low-temperature detectors that were based on similarly sized Li${}_{2}$MoO${}_{4}$ crystals produced from molybdenum with the natural isotopic abundance and molybdenum from the enriched amount in ${}^{100}$Mo [12,13,17,22,23,25]. The Ge light detectors, being gram-scale bolometric devices equipped with smaller NTDs (i.e., reduced heat capacity compared with the Li${}_{2}$${}^{100\mathrm{depl}}$MoO${}_{4}$ bolometers), have an order of magnitude faster response, which is typical for such devices [13,17,22,23,25,48,69].

**Figure 3:**
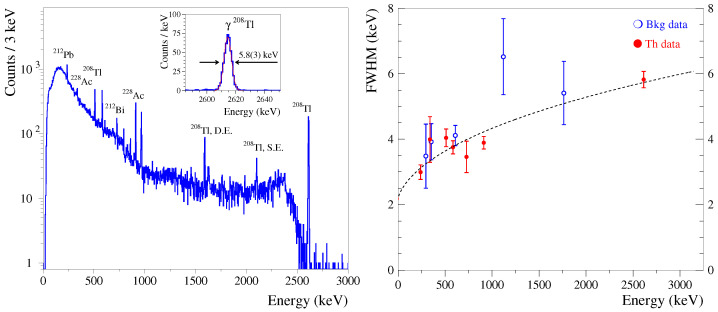
(**Left**) Energy spectrum of a ${}^{232}$Th source, measured using the Li${}_{2}$${}^{100\mathrm{depl}}$MoO${}_{4}$ (LMO-depl-1) bolometer and operated at 12 mK over 125 h. The most intense γ-ray peaks observed in the spectrum are labeled by their origin; D.E. and S.E. mean double and single escape peaks, respectively. A fit to the 2615 keV peak of ${}^{208}$Tl is shown in the inset; the energy resolution is 5.8(3) keV FWHM. (**Right**) The energy dependence of the Li${}_{2}$${}^{100\mathrm{depl}}$MoO${}_{4}$ (LMO-depl-1) bolometer energy resolution in the calibration (red, 125 h) and background (blue, 1109 h) data acquired at 12 mK. The fit is shown by the dashed line.

Then, using an amplitude distribution of the events recorded in the calibration runs, we calibrated the energy scale of the bolometers and evaluated their sensitivity, which was expressed as a voltage amplitude per unit of deposited energy (e.g., nV/keV); we also evaluated the energy resolution of the bolometers in the limit of zero amplitude (baseline noise) and at a mono-energetic radiation. In order to calibrate the Li${}_{2}$${}^{100\mathrm{depl}}$MoO${}_{4}$ bolometers, we used the most intense γ quanta emitted by the ${}^{232}$Th source in the energy interval of 0.2–2.6 MeV, as illustrated in Figure 3 (left). In the background data, we also relied on the presence of γ peaks from environmental radioactivity (mainly the γ-active daughters of radon, ${}^{214}$Pb, and ${}^{214}$Bi; examples are given below). The sensitivities of the Li${}_{2}$${}^{100\mathrm{depl}}$MoO${}_{4}$ bolometers were measured to be ∼20–40 nV/keV (see Table 1); the LMO-depl-1 signal increases by a factor of two at a colder heat sink temperature. Taking into account that the chosen working points are characterized by relatively high NTD currents, the achieved sensitivities are not exceptional among Li${}_{2}$MoO${}_{4}$-based bolometers (the highest reported values are ∼100–150 nV/keV) [12,13,17,22,23,25]. Furthermore, the baseline noise was found to be rather low at ∼2–4 keV FWHM (Table 1), and it was similar to an early reported performance of NTD-instrumented Li${}_{2}$MoO${}_{4}$ bolometers (the best performing detectors have an FWHM noise of ∼1 keV) [12,13,17,22,23,25]. To further improve the baseline noise (e.g., for dark matter search applications), one can reduce the absorber’s volume (i.e., heat capacity) and/or use an advanced performance phonon sensor technology [34]. Despite not being an extraordinary noise resolution, both Li${}_{2}$${}^{100\mathrm{depl}}$MoO${}_{4}$ bolometers show a comparatively high energy resolution, as presented in Figure 3 (right). As it is also seen in Table 1, the energy resolution for high-energy γ quanta (1.8 and 2.6 MeV) is only a factor of 2–3 worse than the resolution at 0 energy, which is a good feature of Li${}_{2}$MoO${}_{4}$ bolometers [13]. Consequently, the Li${}_{2}$${}^{100\mathrm{depl}}$MoO${}_{4}$ energy resolution at the 2615 keV MeV γs, listed in Table 1 and illustrated in Figure 3 (left, inset), is among the best reported for Li${}_{2}$MoO${}_{4}$ low-temperature detectors [13,17,22,23,24,25].

**Figure 4:**
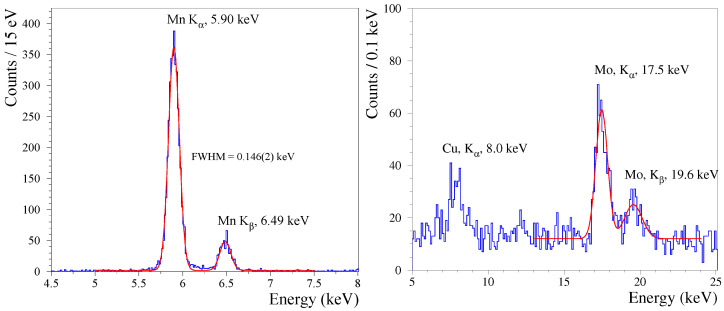
Energy spectra of X-rays from a close ${}^{55}$Fe X-ray source (**left**) and Cu/Mo X-rays induced by the ${}^{232}$Th γ-ray source (**right**), measured by the LD-1-c (1109 h of background data) and LD-2-c (266 h, calibration) bolometers, respectively. Fitting of the spectra using two Gaussians and a linear background component are shown by solid red lines.

Aiming a permanent calibration during measurements, the LD-1-c was supplied by an ${}^{55}$Fe X-ray source, which irradiated a Ge side that was opposite to the LMO-depl-1 crystal. This source emitted a doublet of Mn K${}_{\alpha }$ and K${}_{\beta }$ X-rays with energies of 5.9 and 6.5 keV and intensities 25% and 3%, respectively; an example of the energy spectrum is presented in Figure 4 (left). To overcome the absence of permanent X-ray sources in the assembly of the LMO-depl-2 scintillating bolometer, we irradiated this detector with the ${}^{232}$Th γ source to induce an X-ray fluorescence of the materials that were close to the light detectors, i.e., in the Cu structure and in the crystal. An illustration of the resulting spectrum is shown in Figure 4 (right). Thus, knowing the energy scale, we observed a good sensitivity for two light detectors (1.2–2.2 µV/keV), while the third device had a reduced value (0.4 µV/keV) due to a stronger polarization of the NTD, as exhibited by a lower NTD resistance (see Table 1). This performance is typical for these types of bolometric detectors with NTD thermistors; a further gain is also feasible by reducing the heat capacity of the sensor/absorber (e.g., see [13] and references therein) and/or by upgrading with a Neganov–Trofimov–Luke-effect-based signal amplification [66]. The less sensitive light detector had a comparatively modest noise resolution (about 300 eV FWHM), while the other two detectors demonstrated a rather low noise resolution of 60–100 eV FWHM (e.g., see [4]). The resolution of the 6 keV X-ray peak was found to be close to the baseline noise value, while a more broader 17 keV Mo X-ray peak was detected by both light detectors irrespective of the 5-times difference in the baseline noise. This effect can be explained by a position-dependent response by such thin bolometers [13].

### 3.2. Scintillation Detection and Particle Identification

**Figure 5:**
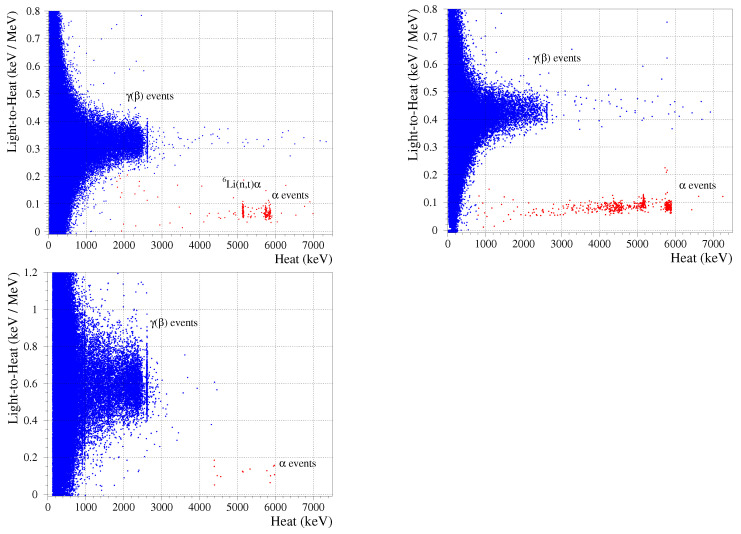
Scintillation (light-to-heat parameter) versus heat energy release measured by the Li${}_{2}$${}^{100\mathrm{depl}}$MoO${}_{4}$ scintillating bolometers. The top panel (**left**) shows a sum of the calibration (125 h) and background (1109 h) data of LMO-depl-1. The LMO-depl-2 events were detected in the background (**top**, **right**; 1536 h) and calibration (**bottom**; 111 h) measurements in coincidence with the bolometric photodetectors LD-2-c and LD-2-s, respectively.

A combination of both heat and scintillation channels of a scintillating bolometer can provide particle identification, which exploits the dependence of the light output on the energy loss mechanism (i.e., particle type) [4,70]. In order to find coincidences between signals in the Li${}_{2}$${}^{100\mathrm{depl}}$MoO${}_{4}$ bolometers and in the associated light detectors, the latter channels were processed using the trigger positions of Li${}_{2}$${}^{100\mathrm{depl}}$MoO${}_{4}$ events and accounting for a difference in the time response (see Table 1), similar to [71]. It is convenient to present such data by normalizing the light detector signal on the corresponding heat energy release, the so-called light-to-heat parameter (L/H), in units of keV/MeV. The dependence of the L/H parameter on the energy and type of particles that impinged the Li${}_{2}$${}^{100\mathrm{depl}}$MoO${}_{4}$ detectors is illustrated in Figure 5.

Several populations of events are clearly seen in each of the data presented in Figure 5. The most dominant one, which is mainly distributed below 3 MeV, is originated by γ(β) particles. We selected γ(β)s with energies above 2 MeV to evaluate the corresponding light-to-heat parameter ($L/H_{\gamma (\beta )}$), which is reported in Table 2. The lowest $L/H_{\gamma (\beta )}$ value (∼0.3 keV/MeV) was obtained for the LMO-depl-1; this is expected due to the sub-optimal scintillation light collection conditions (e.g., the aperture between the crystal and the photodetector, the Cu surrounding instead of it being a reflective foil, the smaller light-detector area). Indeed, the twin detector (LMO-depl-2), which was surrounded by the reflective foil and coupled to the a photodetector with the same size, detected about 30% more scintillation energy (∼0.4 keV/MeV), while the square-shaped light detector allowed almost double the scintillation signal (∼0.6 keV/MeV). At higher energies, such as above ∼3 MeV, we observe populations of events that are characterized by the scintillation being reduced to ∼20% compared with the γ(β)s, as seen in Table 2. These events are originated by αs from either U/Th traces of detector bulk/surface contamination or a U source and α+t particles, the products of neutron capture on ${}^{6}$Li. Despite the different light collection conditions of the characteristics of the Li${}_{2}$${}^{100\mathrm{depl}}$MoO${}_{4}$ scintillating bolometers, the $L/H_{\gamma (\beta )}$ and $QF_{\alpha }$ listed in Table 2 are similar to the ones reported for detectors based on Li${}_{2}$MoO${}_{4}$ crystals produced from molybdenum using the natural isotopic composition as well as molybdenum enriched in ${}^{100}$Mo [13,17,22,23,24,72].

### 3.3. Radiopurity of Li${}_{2}$${}^{100\mathrm{depl}}$MoO${}_{4}$ crystals

**Figure 6:**
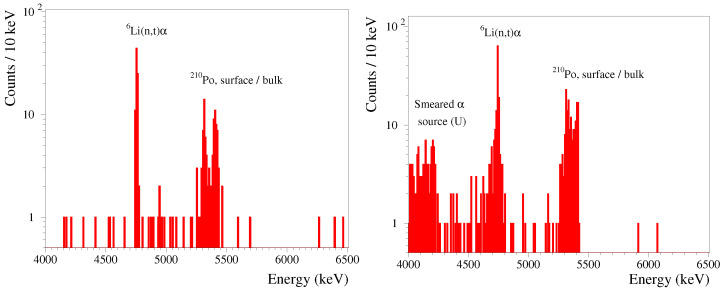
Energy spectra of α events detected by the Li${}_{2}$${}^{100\mathrm{depl}}$MoO${}_{4}$ scintillating bolometers composed of crystals LMO-depl-1 (**left**; 1109 h of measurements) and LMO-depl-2 (**right**; 1528 h), which were operated underground in the C2U facility.

Thanks to efficient particle identification (Figure 5) and comparatively long background measurements, we can investigate the radiopurity of the Li${}_{2}$${}^{100\mathrm{depl}}$MoO${}_{4}$ crystals with a high sensitivity to the α-active radionuclides of the U/Th decay chains. With this aim in mind, we selected α particles from the data of both detectors and re-calibrated the spectra to an alpha-energy scale for an analysis of the α contaminants; the resulting data are shown in Figure 6.

It is seen in Figure 6 that the α spectra of the Li${}_{2}$${}^{100\mathrm{depl}}$MoO${}_{4}$ crystals are rather similar with the exception of the energy region below ∼4.7 MeV, which is populated by α particles from a ${}^{238}$U/${}^{234}$U source that was used in the set-up nearby the LMO-depl-2 detector. The spectra only contain two peak-like structures, which is a clear indication of a high internal radiopurity. Moreover, the first peak at ∼4.8 MeV is originated by the detection of products (α plus triton) of thermal neutron captures by ${}^{6}$Li; this peak is detected by both bolometers at a similar rate of 1.8(2) counts/day. Furthermore, the doublet of 5.3 and 5.4 MeV peaks is a summed contribution of detector surface and crystal bulk contaminations by ${}^{210}$Po; in the later case, an α particle and ${}^{206}$Pb nuclear recoil (taking away 0.1 MeV of energy) were detected. The activity of ${}^{210}$Po in both crystals is the same at ∼35 µBq/kg; it is originated by a residual ${}^{210}$Pb contamination, which is typical for scintillators, particularly for Mo-containing compounds [13,17,73,74,75].

We found no clear evidence of other α-active radionuclides from U/Th chains in the α spectra of the Li${}_{2}$${}^{100\mathrm{depl}}$MoO${}_{4}$ detectors; thus, we set upper limits on their activities in the crystal bulk using the Feldman–Cousins approach [76]. As a signal, we took all events in the range of ±25 keV around the *Q*-value of a radionuclide that we searched for; meanwhile, the background estimate was performed in the neighbor energy interval out of the *Q*-values of the U/Th αs, the process of which is detailed in [73]. The results of the study of the Li${}_{2}$${}^{100\mathrm{depl}}$MoO${}_{4}$ crystals’ radiopurity are summarized in Table 3. The limits on the U/Th activity were obtained on the level of a few µBq/kg; therefore, the radiopurities of the two Li${}_{2}$${}^{100\mathrm{depl}}$MoO${}_{4}$ samples were similar to the purity level of the Li${}_{2}$${}^{100}$MoO${}_{4}$ crystals produced for the LUMINEU [13,15], CUPID-Mo [17,72] and CROSS [23,25] experiments, thanks to the same purification and crystallization protocols applied.

### 3.4. Background Reconstruction Capability of Li${}_{2}$${}^{100\mathrm{depl}}$MoO${}_{4}$ Bolometers

**Figure 7:**
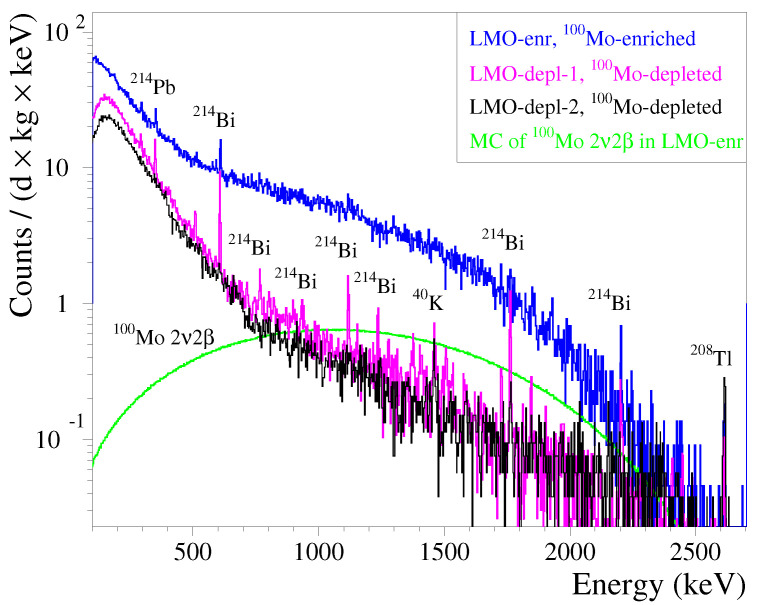
Energy spectra of γ(β) events detected by bolometers based on 0.28 kg lithium molybdate crystals produced from molybdenum that was either depleted in ${}^{100}$Mo (pink, LMO-depl-1, and black, LMO-depl-2) or enriched in ${}^{100}$Mo (blue, LMO-enr data from [23]); the bolometers were operated in the C2U set-up at the Canfranc Underground Laboratory. The detectors LMO-depl-1 and LMO-enr were run together (see Figure 2), while the LMO-depl-2 detector was measured in the next cryogenic run, where the set-up was flushed with deradonized air. It is worth noting that the LMO-enr bolometer was irradiated by a close ${}^{238}$U/${}^{234}$U α source, which emits β particles, ${}^{234}$Th ($Q_{\beta }$ = 0.27 MeV), and ${}^{234m}$Pa ($Q_{\beta }$ = 2.2 MeV). Thus, the difference between the acquired spectra is mainly explained by both the ${}^{100}$Mo two-ν double-β activity (∼3 mHz, $Q_{\beta \beta }$ = 3.0 MeV) and the α-source-induced β background (about 20 mHz of ${}^{234}$Th and ${}^{234m}$Pa) of the Li${}_{2}$${}^{100}$MoO${}_{4}$ detector. A Monte Carlo distribution of the ${}^{100}$Mo two-ν double-β decay events (green) [16] is shown for comparison.

As mentioned in Section 1, ${}^{100}$Mo presents a great interest for double-β decay studies. However, the two-ν double-β decay of ${}^{100}$Mo —which is characterized by the fastest half-life among all double-β active isotopes [41]— is an important source of the background in ν-less double-β decay search experiments. Indeed, it generates a 10 mHz rate in a 1 kg ${}^{100}$Mo-enriched lithium molybdate crystal, and it is a dominant background component in a wide energy interval [13,19,77]. However, even crystals with natural Mo content have a non-negligible internal activity of ${}^{100}$Mo (∼1 mBq/kg). The impact of ${}^{100}$Mo radioactivity can be seen in Figure 7, where the energy spectra accumulated with ${}^{100}$Mo-enriched (up to ∼97%)/depleted Li${}_{2}$MoO${}_{4}$ bolometers in a common measurement at the C2U facility are shown. It is worth noting that the background of the Li${}_{2}$${}^{100}$MoO${}_{4}$ bolometer was spoiled by a β-component of the used external α source with a notable activity, which was around an order of magnitude higher than that of the ${}^{100}$Mo two-ν double-β decay; this is evident in Figure 7. A clear γ background reduction was exhibited by the LMO-depl-2 bolometer in comparison to the LMO-depl-1 data, which was achieved thanks to using a deradonized air flow around the cryostat shielding. Both Li${}_{2}$${}^{100\mathrm{depl}}$MoO${}_{4}$ detectors have a similar high background of below 0.7 MeV, which is explained by a ${}^{210}$Bi activity that is induced by the ${}^{210}$Pb contamination of the lead shield. Moreover, the residual γ(β) activity inside the experimental volume of the set-up, which was detected by both Li${}_{2}$${}^{100\mathrm{depl}}$MoO${}_{4}$ bolometers above ∼1 MeV, is higher than, e.g., the CUPID-Mo [19,77], CUPID-0 [78,79], and CUORE [80,81] experiments. Thus, the difference between the ${}^{100}$Mo two-ν double-β decay distribution and the background data of the Li${}_{2}$${}^{100\mathrm{depl}}$MoO${}_{4}$ detectors, which were acquired in not fully optimized background conditions, is not remarkable as it may have been in a better shielded set-up; see Figure 7.

It is evident in Figure 7 that the Li${}_{2}$${}^{100\mathrm{depl}}$MoO${}_{4}$ bolometer allows for a significantly improved reconstruction of the γ background, including low-intensity contributions, compared with the Li${}_{2}$${}^{100}$MoO${}_{4}$ detector, which has a dominant double-β (and β) decay events continuum. Therefore, Li${}_{2}$${}^{100\mathrm{depl}}$MoO${}_{4}$ low-temperature detectors with high spectrometric performance and high radiopurity can provide complementary information about the background model in double-β decay searches with Li${}_{2}$${}^{100}$MoO${}_{4}$ bolometers. A similar example is the CUPID-0 experiment with two natural and twenty-four ${}^{82}$Se-enriched zinc selenide bolometers [48]); however the detectors using selenium at the natural isotopic concentration were not included into the background model analysis [78]. Moreover, a combination of Li${}_{2}$${}^{100\mathrm{depl}}$MoO${}_{4}$ and Li${}_{2}$${}^{100}$MoO${}_{4}$ bolometers of similar performance and purity would allow for the extraction of the half-life and spectral shape of the ${}^{100}$Mo two-ν double-β decay. A similar approach has been used in several double-β searches, such as in ${}^{40}$Ca-/${}^{48}$Ca-enriched calcium fluoride scintillation detectors [82], a ${}^{78}$Kr-enriched/depleted gas filled proportional counter [83], and a ${}^{136}$Xe-enriched/depleted xenon gas-based time projection chamber [84]. However, the enrichment in ${}^{48}$Ca, which is present in natural calcium at a ∼0.2% level, is not yet available in as large of quantities as for ${}^{100}$Mo, ${}^{78}$Kr, and ${}^{136}$Xe [85]. Moreover, the very long half-lives of ${}^{78}$Kr (10${}^{22}$ yr) and ${}^{136}$Xe (10${}^{21}$ yr) require a huge exposure to collect reasonably high datapoints for double-β decay events; the rate of ${}^{100}$Mo being more than two orders of magnitude faster is an advantage for this type of study.

In the case of a resonant absorption of solar axions to the first excited state of ${}^{7}$Li along with its subsequent γ de-excitation, a background in the Li${}_{2}$${}^{100\mathrm{depl}}$MoO${}_{4}$ bolometer at ∼0.5 MeV that is an order of magnitude lower than the Li${}_{2}$${}^{100}$MoO${}_{4}$ one would provide a higher sensitivity to an expected peak at 478 keV [11,38]. At lower energies, the contribution of the ${}^{100}$Mo two-ν double-β decay becomes negligible [23], causing no preference in ${}^{100}$Mo content in the Li${}_{2}$MoO${}_{4}$ crystals during the search of a spin-dependent dark matter interaction on ${}^{7}$Li. At the same time, the presence of ${}^{6}$Li allows for neutron detection (illustrated above in Figure 5 and Figure 6); this can be exploited for neutron flux monitoring, which is particularly relevant for dark matter search applications.

## 4. Conclusions

In the present study, we demonstrated that scintillating bolometers composed of lithium molybdate crystals produced from molybdenum depleted in ${}^{100}$Mo (Li${}_{2}$${}^{100\mathrm{depl}}$MoO${}_{4}$) show a high performance that is comparable with the devices that are based on crystals from molybdenum of the natural isotopic composition or molybdenum enriched in ${}^{100}$Mo. Thanks to the strict purification and crystallization protocols, which were developed and already applied to high-sensitivity searches for ${}^{100}$Mo ν-less double-β decay, the radiopurity of Li${}_{2}$${}^{100\mathrm{depl}}$MoO${}_{4}$ crystals is rather high and is comparable to ${}^{100}$Mo-enriched crystals of the same production line. Thus, given an availability of molybdenum that is depleted in ${}^{100}$Mo (a by-product of industrial enrichment in ${}^{100}$Mo) for Li${}_{2}$${}^{100\mathrm{depl}}$MoO${}_{4}$ crystals production, in addition to having a high spectrometric performance, efficient scintillation-assisted particle identification capability, and high material radiopurity, Li${}_{2}$${}^{100\mathrm{depl}}$MoO${}_{4}$ scintillating bolometers show a high potential for applications in rare-event search experiments. In particular, such detectors represent a great interest for studies of ${}^{100}$Mo two-ν double-β decay, searches for ${}^{7}$Li axions and spin-dependent interactions of dark matter particles on ${}^{7}$Li, and ${}^{6}$Li-based neutron spectroscopy and γ(β) background control measurements in low-temperature, low-background experiments. Given the obtained results, we will use the available Li${}_{2}$${}^{100\mathrm{depl}}$MoO${}_{4}$ crystals in the CROSS experiment and propose exploiting such low-temperature detectors in CUPID.

## Figures and Tables

**Table 1:** Performance of Li${}_{2}$${}^{100\mathrm{depl}}$MoO${}_{4}$ scintillating bolometers and Ge light detectors. We report the detector plate temperature, NTD resistance, signal rise and decay time parameters, detector sensitivity, baseline noise resolution, and energy resolution at a given energy.

Bolometer	Temperature	Resistance	Rise	Decay	Sensitivity	FWHM_*Noise*_	FWHM (keV)
		of NTD	Time	Time	(nV/keV)	(keV)	at Energy (keV)
	(mK)	(MΩ)	(ms)	(ms)			
LMO-depl-1	18	2.4	16	112	17	3.66 (3)	5.9 (10) at 1765
	12	6.5	20	115	37	2.18 (3)	5.8 (3) at 2615
LMO-depl-2	14	3.0	16	97	29	3.80 (3)	6.8 (3) at 2615
LD-1-c	18	1.6	1.5	9.0	1200	0.097 (1)	0.174 (4) at 5.9
	12	2.6	1.6	10.5	1380	0.100 (1)	0.146 (2) at 5.9
LD-2-s	14	0.47	1.6	5.2	380	0.343 (5)	0.94 (6) at 17.5
LD-2-c	14	4.4	2.0	7.8	2200	0.059 (1)	0.90 (6) at 17.5

**Table 2:** Results of the scintillation detection using the Li${}_{2}$${}^{100\mathrm{depl}}$MoO${}_{4}$ scintillating bolometers. We report the light-to-heat ratios for γ(β) events ($L/H_{\gamma (\beta )}$) and the quenching factors for the scintillation induced by αs of ${}^{210}$Po ($QF_{\alpha }$).

Crystal	Photodetector	$L/H_{\gamma (\beta )}$ (keV/MeV)	${\mathrm{QF}}_{\alpha }$
LMO-depl-1	LD-1-c	0.33 (3)	0.21 (4)
LMO-depl-2	LD-2-c	0.44 (3)	0.19 (4)
	LD-2-s	0.59 (9)	

**Table 3:** Radioactive contamination of the Li${}_{2}$${}^{100\mathrm{depl}}$MoO${}_{4}$ crystals by α-active radionuclides from ${}^{238}$U/${}^{232}$Th families (their *Q*-values are listed in keV). The uncertainties are given at a 68% C.L., while the limits are set at 90% C.L.

Crystal	Activity (µBq/kg)					
	${}^{232}$Th	${}^{228}$Th	${}^{238}$U	${}^{234}$U	${}^{226}$Ra	${}^{210}$Po
	[4082]	[5520]	[4270]	[4858]	[4871]	[5407]
LMO-depl-1	<2	<2	<2	<5	<7	35 (6)
LMO-depl-2		<2			<4	36 (5)

## Data Availability

Data are available upon reasonable request.
